# Conservative management of asymptomatic vertebral artery stent fracture with fragment embolisation to V3 segment: a case report

**DOI:** 10.3389/fsurg.2025.1659557

**Published:** 2025-09-18

**Authors:** Bin Hou, Chuangzhong Li, Yuan Cao, Xiaogang Wang, Dandan Gao, Ligang Chen

**Affiliations:** ^1^ Department of Neurosurgery, Shandong University of Traditional Chinese Medicine Affiliated Hospital, Jinan, China; ^2^ Department of Neurosurgery, Liaoning University of Traditional Chinese Medicine Affiliated Hospital, Shenyang, China; ^3^ Department of Neurosurgery, General Hospital of the Northern Theater Command, Shenyang, China

**Keywords:** vertebral artery origin, stenosis, stent fracture, complication, atherosclerosis

## Abstract

**Introduction:**

Stent fracture represents a recognised but rare complication tied to vertebral artery origin stenting.

**Methods:**

We present a male who was diagnosed with left vertebral artery origin stenosis due to vertigo and diplopia, and who was deployed with a rapamycin-eluting stent. One-year follow-up computed tomography angiography demonstrated mid-stent fracture with distal fragment migration into the V3 segment, but the patient was asymptomatic and managed medically with close surveillance.

**Results:**

This case elucidates that rapamycin-eluting stents are an effective treatment for stenosis at the vertebral artery origin.

**Discussions:**

In rare instances involving complete stent fracture and migration of distal debris, conservative management may be a feasible option for asymptomatic patients.

## 1 Introduction

Vertebral artery (VA) origin stenosis constitutes approximately 30% of posterior circulation strokes, and stenting may be warranted for symptomatic patients with ≥50% stenosis refractory to antiplatelet treatment (1). Stent fracture (SF) is a recognised but rare complication of VA stenting, and comprehensive data remain scarce. Herein, we present an asymptomatic case of VA-origin SF with distal fragment migration into the V3 segment. Written informed consent for publication was obtained from the patient, and the case was reviewed by the ethics committee.

## 2 Case

**Figure 1:**
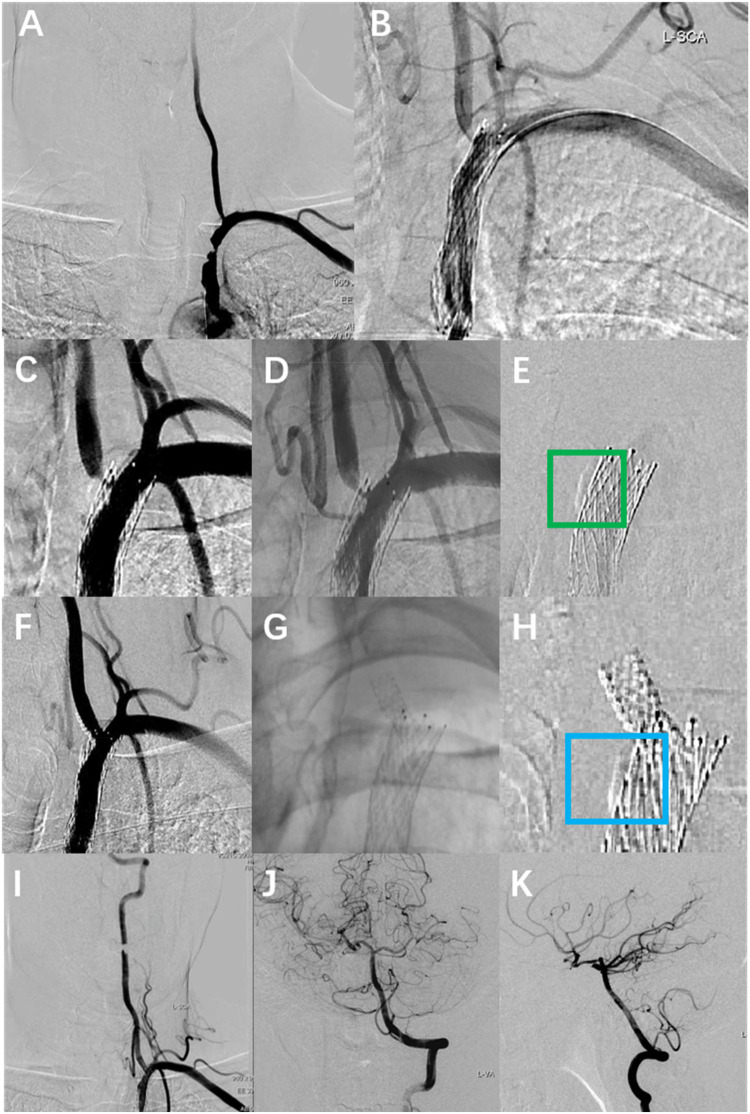
The DSA images of the patient's multiple intravascular treatments. **(A)** Preoperative DSA showed severe stenosis of the left SCA and mild stenosis of the left VA origin. **(B)** Left SCA placement of Protégé RX (8 mm × 40 mm, eV3), with the distal portion of the stent covering the VA origin. **(C,D)** Re-angiography was performed eight months after the SCA stenting with severe stenosis at the left VA origin. **(E)** Calcified plaque at the left vertebral-subclavian artery junction (green box). **(F,G)** A rapamycin-eluting stent (4 mm × 12 mm, MauroraStent, Salubris) was placed at the VA origin, overlapping with the left SCA stent. **(H)** Calcified plaque is located proximal to the VA stent (blue box). **(I–K)** The whole left VA is unobstructed after the left VA stenting.

A 56-year-old male patient with a history of hypertension and dyslipidemia, previously untreated. He was admitted to our hospital after he experienced persistent vertigo and intermittent diplopia. Digital subtraction angiography (DSA) demonstrated the right internal carotid artery (ICA) occlusion, severe stenosis at the bifurcation of the left common carotid artery (CCA) (about 71%), left subclavian artery (SCA) severe stenosis (about 82%) with steal phenomenon and the left VA origin mild stenosis (Figure 1A). Additionally, the posterior circulation supplies the anterior circulation via the left posterior communicating artery. Considering symptoms mainly from the SCA steal, the Protégé RX (8mm × 40 mm, eV3) was deployed (distal edge partially covering the VA origin). (Figure 1B). Postoperatively, symptoms markedly resolved, and he routinely used antiplatelet medications, with Aspirin (100 mg/d) for a lifetime and Hydrochloric acid hydrochlorothiazide (75 mg/d) for 3 months. In addition, taking nifedipine controlled-release tablets (30 mg/d) and atorvastatin calcium tablets (20 mg/d) helped maintain normal blood pressure and lipid levels. Two months later, the left CCA endarterectomy was performed.

Eight months after stenting for SCA, the patient experienced recurrent vertigo, prompting a reevaluation through repeat angiography, which demonstrated the severe stenosis at the left VA origin. (Figures 1C,D). Then the revascularisation was conducted through the femoral access: deployment of a rapamycin-eluting stent (4 mm × 12 mm, MauroraStent, Salubris) at the VA origin following dilation (6 atm), overlapping the left SCA stent, resulting in an RSR of 5% (Figures 1E–K). After stenting, the symptoms of this patient improved. He persisted in the administration of antiplatelet medications, including Aspirin (100 mg/d) for a lifetime and hydrochloric acid hydrochlorothiazide (75 mg/d) for 3 months after the VA stenting. Lipid-regulating and antihypertensive medications remained unaltered.

**Figure 2:**
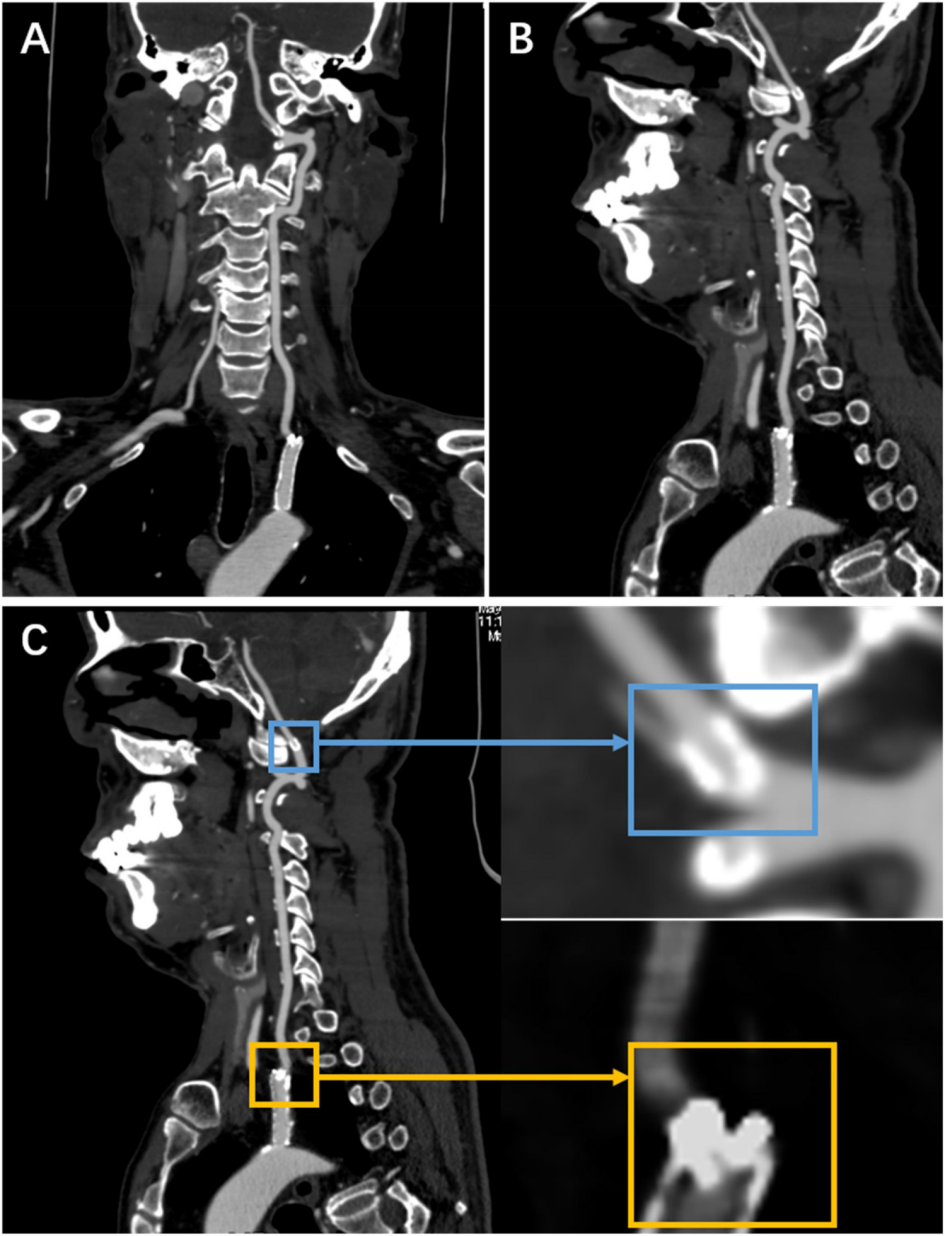
The CTA was done a year following stenting of the left VA origin. **(A,B)** They show a stent fracture in the centre, with the distal fragment migrating to the V3 segment. **(C)** It shows a local magnification of both fragments (the yellow rectangle indicates the proximal fragment, the blue rectangle represents the distal fragment).

**Figure 3:**
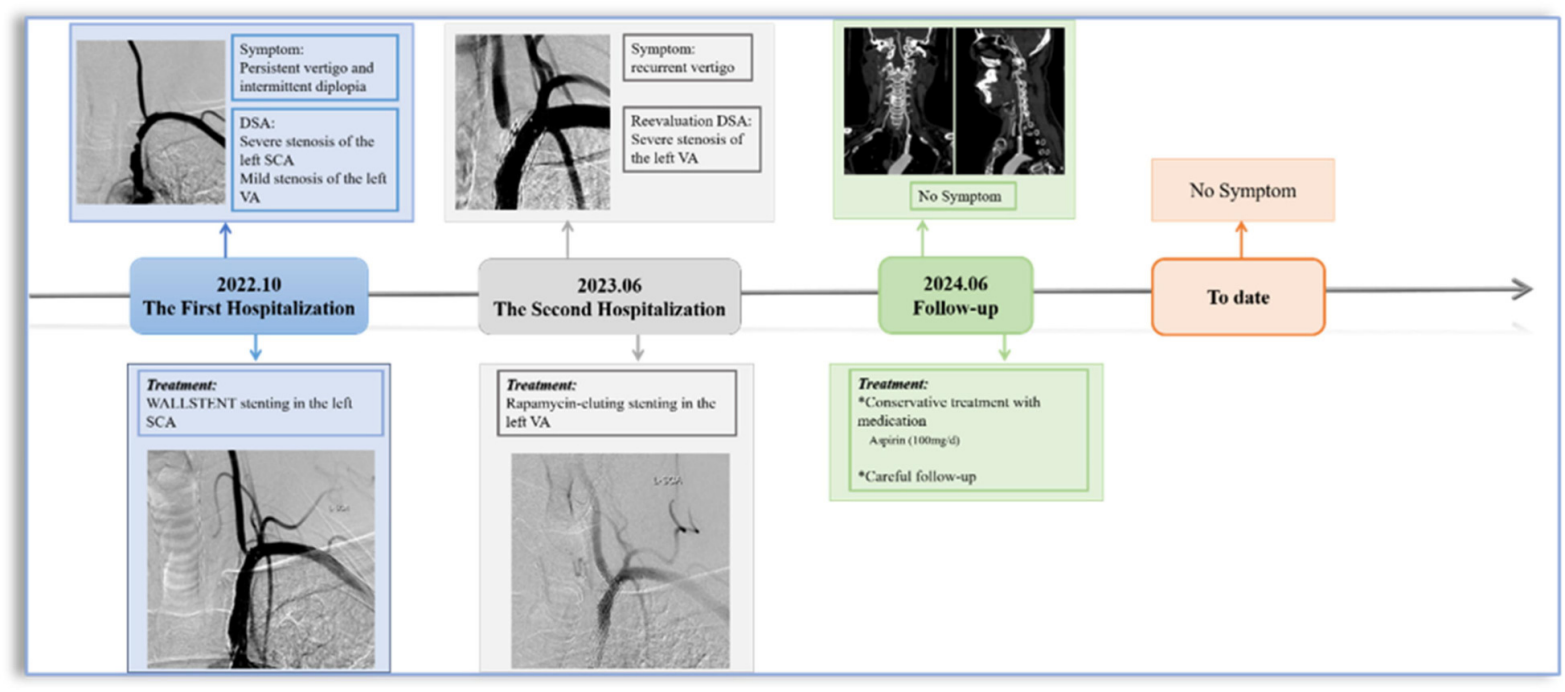
The timeline for this patient.

Surveillance CTA performed one year following the VA stenting revealed the mid-stent fracture with distal fragment migration to the V3 segment, and both stent debris remained patent with no pseudoaneurysm (Figure 2). Then, we strongly recommend the patient undergo DSA to assess the vascular condition accurately. However, he was asymptomatic and was experiencing psychological distress from multiple previous neurointerventional procedures, so he declined to undergo the DSA examination. Finally, respecting patient autonomy, after the detailed discussion, we opted for maintaining antiplatelet therapy, Aspirin (100 mg/d), with close observation. To date, he is without symptoms. Figure 3 shows the timeline of the complete illness course of this patient.

## 3 Discussion

SF, a recognised complication in peripheral arteries (2), is infrequently documented at the VA origin. This uncommon instance details an asymptomatic stent fracture originating from the vertebral artery, with a distal fragment migrating into the V3 segment. To our knowledge, no such occurrences have been recorded.

The genuine incidence of SF in the VA origin remains ambiguous. Li et al. (3) reported a 30% 5-year SF occurrence (*n* = 17). Conversely, Wang et al.'s 6-month prospective study involving 104 stents in the VA origin discovered no fractures (4). These findings indicate SF may be underestimated due to the insufficiency of extensive prospective studies.

Uncertainty surrounds the contributing justifications to SF, which could encompass the curvature of the VA origin, lesion characteristics, stent attributes, and procedural methods (4, 5). Stents implanted in flexible VA ostia, especially in curved segments, undergo mechanical stress due to vessel recoil towards native anatomy, which facilitates SF. **Both coronary artery and lower limb artery stent fractures are associated with mechanical stress (**Table 1**). Coronary artery stent fractures are primarily caused by the periodic stress generated by cardiac contractions, while lower limb artery stent fractures are closely related to joint movement and muscle activity.** Tang et al. (6) identified that respiratory-induced dynamic VA tortuosity correlated with SF: both fractures in their 178-patient cohort occurred at sites of respiratory motion. This repetitive stress modifies stent configuration, leading to metal fatigue and SF. In previous studies, calcified coronary plaque may compromise stent structural integrity during implantation, potentially contributing to SF (7). The patient exhibited calcified plaques at the stenotic vertebral artery origin (Figures 1E,H). Therefore, we consider that major curvature and calcified plaque could necessitate alternative therapeutic approaches such as drug-coated balloon angioplasty and surgical VA ostium transplantation, which may potentially offer comparable clinical benefits to patients.

**Table 1:** Characteristics of SF patients.

No.	Position	SF time (month/m)	Clinical manifestations after SF	Restenosis OR occlusion	Pseudoaneurysm (Yes/No)	Treatment
Peripheral arteries						
1 (10)	Popliteal artery	12 months	Pain intermittent claudication	Restenosis	No	Surgery to remove stent debris and revascularization by patch
	Femoral artery	12 months	Pain	Occlusion	No	Endovascular revascularization
2 (11)	Popliteal artery	18 months	Pain intermittent claudication	No	Yes	Endovascular revascularization
3 (12)	Femoral and popliteal artery	24 months	Intermittent claudication	Occlusion	Yes	endovascular revascularization
Intracranial arteries						
4 (13)	Middle cerebral artery	7 months	No	Occlusion	No	Medication with follow-up
Coronary arteries						
5 (14)	Coronary artery	180 months	Chest pain/pressure	Restenosis	Yes	Endovascular revascularization
6 (15)	Coronary artery	168 months	Unstable angina	Restenosis	Yes	Endovascular revascularization
7 (16)	Coronary artery	3 months	Chest pain	No	Yes	Bypass graft surgery

The keyword “complete stent fracture” was used to search the PubMed and Web of Science databases for relevant research (2015–2025). After removing studies that did not offer full details, seven papers reported stent fractures with displacement in peripheral arteries, intracranial arteries, and coronary arteries.

Stent properties may correlate with fracture. Rapamycin-eluting stents mitigate restenosis through antiproliferative effects, which may elevate SF risk in coronary applications (8) due to compromised structural integrity for drug embedding. This may extend to VA origin stenting. Open-cell stent designs appear more fracture-prone due to fewer connection points despite enhanced flexibility, and cervical motion and ventilation may exacerbate structural deformation; however, these conjectures require further verification.

In this case, prior placement of the SCA stent partially obstructed the VA ostium with the perpendicular configuration, potentially explaining the rapid progression of stenosis (Figures 1C,D). Frictional contact between the unreleased VA stent and SCA stent during implantation likely induced microcracks at their interaction point, potentially causing SF. Moreover, previous analysis of coronary arteries has proved that parallel and overlapping stents exhibit stress concentration zones, where pulsatile flow induces cutting effects, leading to a gradual accumulation of SF (9). Therefore, analogous stress mechanisms may participate in the perpendicular configuration in this case (Figures 1F,G).

No standardised treatment exists for SF. For restenosis or symptomatic patients, stent-in-stent deployment is an alternative. Conservative management may be appropriate for asymptomatic patients. Table 1 details the clinical characteristics of stent fractures by vascular location. It shows that symptomatic patients underwent surgery while asymptomatic patients received conservative treatment. However, insufficient evidence was found due to the limited number of included studies. **Despite V3 segment fragments in this case, the absence of ischemic symptoms and luminal stenosis warranted conservative therapy with careful monitoring. If imaging studies during follow-up confirm significant displacement of the distal fragment or associated stenosis, consider placement of a self-expanding stent as necessary.** The patient remains asymptomatic to date.

## 4 Conclusion

SF with distal fragment displacement in the VA origin is a rare occurrence. In cases necessitating surgical intervention for VA origin stenosis characterised by calcified plaques, severely twisted vessels, or proximity to the SCA stent, drug-coated balloon angioplasty may serve as an alternative treatment option to mitigate the risk of SF. For asymptomatic patients, conservative management with close monitoring may be considered viable. Nonetheless, as this report pertains to a singular case, further prospective studies are required to determine optimal treatment strategies.

## Data Availability

The original contributions presented in the study are included in the article/Supplementary Material, further inquiries can be directed to the corresponding author.

## References

[1] Chen H Colasurdo M Costa ML Kan P. Endovascular management of extracranial vertebral artery stenosis. J Neurointerv Surg. (2025) 17:1–5. 10.1136/jnis-2024-022618 39922695

[2] Bellissard A Kuntz S Lejay A Chakfé N. Systematic review of femoral artery stent fractures. EJVES Vasc Forum. (2024) 62:48–56. 10.1016/j.ejvsvf.2024.08.001 39328303 PMC11426108

[3] Li MKA Tsang ACO Tsang FCP Ho WS Lee R Leung GKK Long-term risk of in-stent restenosis and stent fracture for extracranial vertebral artery stenting. Clin Neuroradiol. (2019) 29(4):701–6. 10.1007/s00062-018-0708-y 30039353

[4] Ma G Song L Ma N Raynald, Shuai J Wu W Safety and efficacy of rapamycin-eluting vertebral stents in patients with symptomatic extracranial vertebral artery stenosis. Front Neurol. (2021) 12:649426. 10.3389/fneur.2021.649426 34899552 PMC8662782

[5] Vértes M Nguyen DT Székely G Bérczi Á Dósa E. The incidence and risk factors of stent fracture in patients treated for proximal common carotid artery stenosis. J Vasc Surg. (2020) 71(3):824–31.e1. 10.1016/j.jvs.2019.04.492 31405760

[6] Tang X Tang F Hu C Wang Q Long W Li L. Dynamic respiratory tortuosity of the vertebral artery ostium. J Endovasc Ther. (2017) 24(1):124–9. 10.1177/1526602816676254 27831484

[7] McInerney A Travieso A Jerónimo Baza A Alfonso F Del Val D Cerrato E Impact of coronary calcium morphology on intravascular lithotripsy. EuroIntervention. (2024) 20(10):e656–e68. 10.4244/eij-d-23-00605 38776142 PMC11100505

[8] Modolo R Chichareon P Kogame N Asano T Chang CC de Winter RJ A prospective multicentre randomised all-comers trial to assess the safety and effectiveness of the thin-strut sirolimus-eluting coronary stent supraflex: rationale and design of the thin strut sirolimus-eluting stent in all comers population vs everolimus-eluting stent (talent) trial. EuroIntervention. (2019) 15(4):e362–e9. 10.4244/eij-d-18-00499 30066672

[9] Kennedy SM Vasanthanathan A Jeen Robert RB Vignesh Moorthi Pandian A. Impact of mechanical engineering innovations in biomedical advancements. In Vitro Model. (2024) 3(1):5–18. 10.1007/s44164-024-00065-4 39872067 PMC11756506

[10] Wittig T Steiner S Schmidt A Scheinert D Branzan D. Popliteal artery entrapment syndrome: a rare cause of interwoven nitinol stent fracture after femoropopliteal interventions. JACC Case Rep. (2022) 4(7):424–8. 10.1016/j.jaccas.2021.12.027 35693898 PMC9175198

[11] Dos Reis JMC Kudo FA do Carmo Bastos M. Fracture of a popliteal nitinol stent and pseudoaneurysm: a case report and review of the literature. J Surg Case Rep. (2019) 2019(11):rjz312. 10.1093/jscr/rjz312 31737245 PMC6847879

[12] Lee YJ Shin DH Kim JS Kim BK Ko YG Hong MK Femoropopliteal artery stent fracture with recurrent in-stent reocclusion and aneurysm formation: successful treatment with self-expandable viabahn endoprosthesis. Korean Circ J. (2015) 45(6):522–5. 10.4070/kcj.2015.45.6.522 26617656 PMC4661369

[13] Gellen JS Pfaff JAR. Mid-Term follow-up of a fractured flow diverter in the internal carotid artery. Radiol Case Rep. (2025) 20(6):2878–81. 10.1016/j.radcr.2025.02.091 40224230 PMC11987566

[14] Takeda M Shiba N. Non-invasive recanalization of first-generation sirolimus-eluting stent thrombosis due to stent fracture and coronary artery aneurysm after clopidogrel treatment 15 years after implantation. J Cardiol Cases. (2024) 29(5):209–13. 10.1016/j.jccase.2024.01.004 39100514 PMC11295020

[15] Fujita T Tazaki J Toyofuku M. A case report of coronary artery aneurysms with restenosis and stent fractures developed 14 years after sirolimus eluting stents implantation successfully treated with drug-coated balloons. Eur Heart J Case Rep. (2024) 8(2):ytae050. 10.1093/ehjcr/ytae050 38332918 PMC10852020

[16] Rusali CA Cojocaru L Șerbanescu IC Scupra V Iurco D. Case report: intracoronary stent fracture complicated with coronary abscess and fistulization into the pericardium. Eur Heart J Case Rep. (2024) 8(10):ytae532. 10.1093/ehjcr/ytae532 39450322 PMC11500811

